# Benign cutaneous plexiform hybrid tumor of perineurioma and cellular neurothekeoma arising from the nose

**DOI:** 10.1186/1746-1596-8-165

**Published:** 2013-10-08

**Authors:** Sohsuke Yamada, Shohei Kitada, Atsunori Nabeshima, Hirotsugu Noguchi, Yasuyuki Sasaguri, Masanori Hisaoka

**Affiliations:** 1 Department of Pathology and Cell Biology, University of Occupational and Environmental Health, 1-1 Iseigaoka, Kitakyushu, Yahatanishi-ku 807-8555, Japan; 2 Department of Pathology and Oncology, School of Medicine, University of Occupational and Environmental Health, 1-1 Iseigaoka, Kitakyushu, Yahatanishi-ku 807-8555, Japan

**Keywords:** Benign cutaneous plexiform hybrid tumor of perineurioma and cellular neurothekeoma, Nose

## Abstract

Very recently, Requena *et al.* have demonstrated the detailed clinicopathological features of 9 cases of a benign cutaneous plexiform nerve sheath tumor with hybrid characteristics of perineurioma and cellular neurothekeoma, given the name as a benign cutaneous plexiform hybrid tumor of perineurioma and cellular neurothekeoma, all of which were peculiarly located on the lips. Herein we described the first case of that arising from the nose, but not the lip, representing a histological hybridoma of perineurioma and cellular neurothekeoma after thorough consideration especially with its immunohistochemical profile.

## Letter to the editor

There have been several interesting reports describing hybrid peripheral nerve sheath tumors comprising of biphasic (i.e., hybrid) features of neoplastic cells, as follows: a mixture of neurofibroma and schwannoma; schwannoma and perineurioma; perineurioma and granular cell tumor; and so on [1-4]. More recently, Requena *et al.* showed the detailed clinicopathological features of 9 cases of a benign cutaneous plexiform nerve sheath tumor with hybrid characteristics of perineurioma and cellular neurothekeoma, given the name as a benign cutaneous plexiform hybrid tumor of perineurioma and cellular neurothekeoma (**HPN**), all of which were peculiarly located on the lips [1]. Herein we reported an extremely rare and first case of **HPN** of the nose occupying the dermis to superficial subcutis as a well-demarcated nodule.

**Figure 1:**
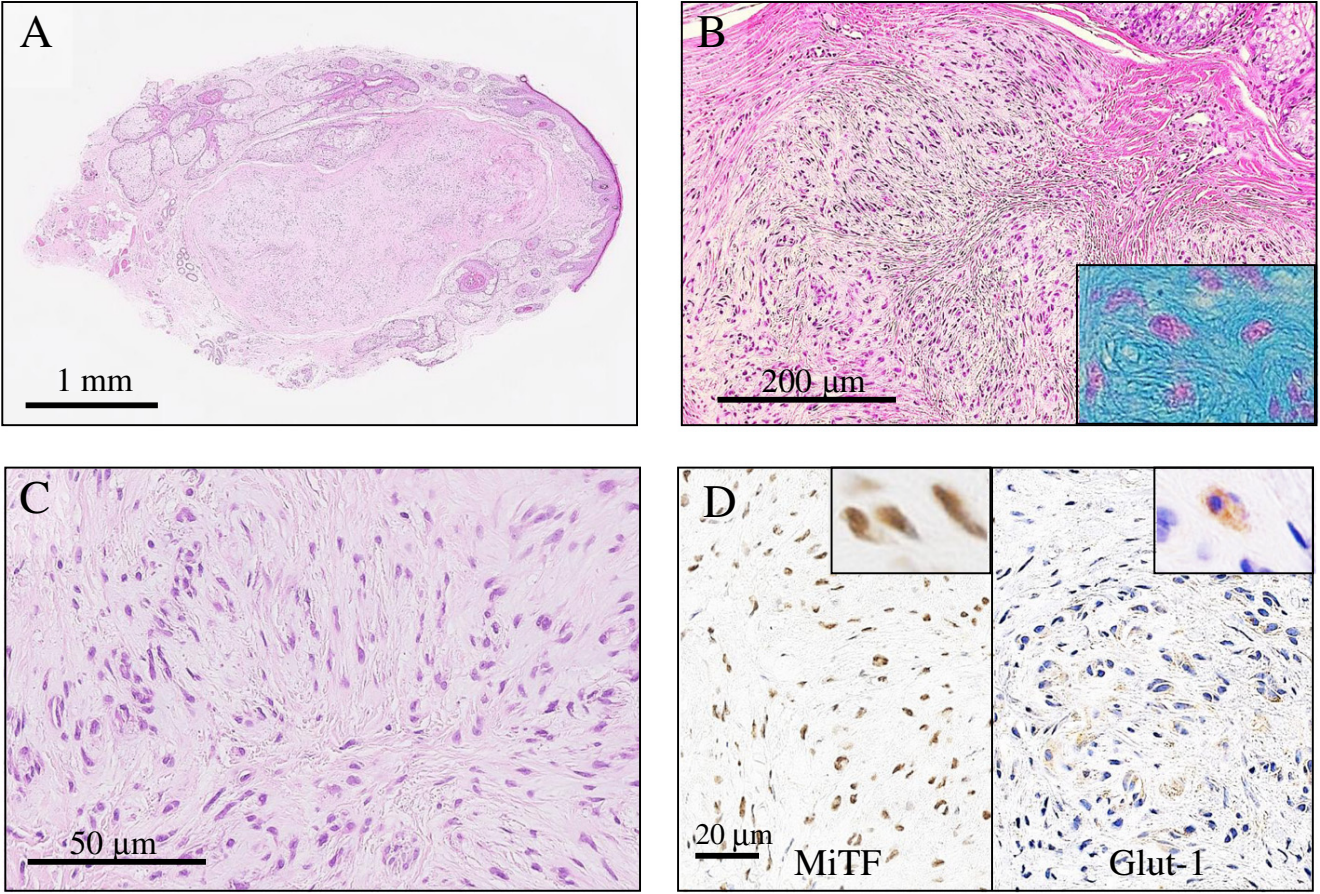
**Microscopic and immunohistochemical examination of the resected HPN specimen. (A)** A scanning magnification of **HPN** (H&E stains) showed well-circumscribed but uncapsulated dermal to superficial subcutaneous multi-lobulated nodular lesions in a plexiform manner, separated by a slightly dense and sclerotic collagenous stroma, and compressing the pre-existing sebaceous glands. Bar = 1 mm. **(B)** Its multi-lobulated parts (H&E stains) were composed of a proliferation of neoplastic cells, arranged in tiny round or whorled nests and embedded in a relatively abundant Alcian blue-positive myxoid stroma (inset). Bar = 200 μm. **(C)** High-power view demonstrated that most of these neoplastic cells had a small and oval to plump spindle shape, with vesicular nuclei, inconspicuous nucleoli, abundant pale eosinophilic cytoplasm, and indistinct cellular borders (H&E stains). Mitotic figures were very rarely encountered. Bar = 50 μm. **(D)** In immunohistochemistry, the tumor cells of **HPN** were specifically positive (insets) for MiTF (lt.) and Glut-1 (rt.). Bars = 20 μm.

The patient presented here, a 30-year-old female with an unremarkable previous medical history, had a 2-year history of a gradually enlarging painless and red-brownish solitary dome-shaped papule in the right wing of the nose. Dermatologists first interpreted it as a benign subcutaneous tumor, and a simple excision was performed. Gross examination revealed a fairly well-demarcated and elastic hard nodular lesion in the dermis, measuring approximately 5 × 3 mm in diameter. On scanning magnification, the tumor consisted of a well-circumscribed but uncapsulated dermal to superficial subcutaneous multi-lobulated nodular lesion in a plexiform fashion, separated by a slightly dense and sclerotic collagenous stroma, and compressing the pre-existing sebaceous glands (Figure 1A). Excision was diagnosed as complete by this histopathological examination. The covering epidermis showed no remarkable change. Microscopically, its multi-lobulated parts were composed of a proliferation of neoplastic cells arranged in tiny round or whorled nests, embedded in a relatively abundant Alcian blue-positive myxoid stroma (Figure 1B) but not in a prominent sclerotic background. On high-power view, most of these neoplastic cells revealed a small and oval to plump spindle shape, having vesicular nuclei, inconspicuous nucleoli, abundant pale eosinophilic cytoplasm, and indistinct cellular borders (Figure 1C). Mitotic figures were very rarely seen. Immunohistochemically, the neoplastic cells were negative for S-100 protein, EMA, cytokeratin, CD34, GFAP, α-SMA, desmin, and claudin-1, whereas strongly positive for vimentin, specifically positive for MiTF (Figure 1D), NKI/C3, Glut-1 (Figure 1D), PGP9.5, CD10, and NSE, and weakly positive for CD68 and CD99. By contrast, the MIB-1 (Ki67) labeling index was noted in much less than 1% in the tumor cells. All immunohistochemical profile of these neoplastic cells is summarized in Table 1. Based on all these features, since it is suggested that the present tumor comprises of hybrid elements of cellular neurothekeoma and perineurioma, we finally made a diagnosis of **HPN** in the right wing of the nose. To date, approximately one year routine follow-up after the surgery is established, and the patient remains well and no recurrence has been recognized.

**Table 1:** Immunohistochemical profile of the neoplastic cells in our case of HPN located on the nose

**Positive**	**Negative**
MiTF	S-100 protein
NKI/C3	EMA
Glut-1	AE1/AE3
Vimentin	Claudin-1
CD 10	GFAP
CD99	α-SMA
PGP 9.5	Desmin
NSE	CD34
CD68	

First, we should insist that the current case report of HPN arising from the wing of the nose is not new with the exception of the location. Our case unusually occurred in the nose, but not the lip. Actually, Requena *et al.* have reported that **HPN** seems to have a special predilection for perioral skin, however, its etiology remains to be elucidated [1]. All pathologists should be aware that its histologically, rather than clinically, characteristic findings from extensively careful immunohistochemical examination can induce one of differential diagnoses, and possibly a correct diagnosis. **HPN** of the skin, including not only the face but the extremities and trunk, may be more common than generally considered. It would be intriguing to study this topic after collecting and investigating many cases of **HPN**.

The present case remarkably shares several histolpathological and immunohistochemical features with perineurioma and cellular neurothekeoma, respectively. Pathological differential diagnoses of our case would include perineurioma, cellular neurothekeoma, nerve sheath myxoma (classic neurothekeoma), desmoplastic neurothekeoma, superficial angiomyxoma (cutaneous myxoma), solitary neurofibroma with prominent differentiation of Meissner bodies, or spindle cell carcinoma. In fact, the 3 neoplasms of **HPN**, perineurioma, and cellular neurothekeoma share the morphological features, displaying a plexiform proliferation of plump spindle or oval neoplastic cells arranged in a nested or whorled fashion and embedded in a myxoid stroma, that should be completely absent in desmoplastic neurothekeoma and superficial angiomyxoma [1,5,6]. The diagnoses of nerve sheath myxoma, neurofibroma, and spindle cell carcinoma also can be simply excluded out, based on immunohistochemical examination, since those tumors typically expresses S-100 protein, CD34 and S-100 protein, and cytokeratin, respectively, but the current case not [7-9]. Furthermore, the tumor cells in our case overtly shares with cellular neurothekeoma the specific immunoexpression for MiTF, NKI/C3, PGP9.5, and NSE [1,8], whereas shares with perineurioma the immunoexpression for Glut-1, CD10, and PGP9.5 and, although to a lesser intensity, the positivity for CD99 [1,8]. Immunohistochemical characteristics of the 3 neoplasms of our HPN, perineurioma, and cellular neurothekeoma are summarized in Table 2. Therefore, our final diagnosis is the first case of **HPN** on the nose, but not on the lip, representing a histological hybridoma of perineurioma and cellular neurothekeoma after thorough consideration especially with its immunohistochemical profile, even though our initial diagnosis was merely cellular neurothekeoma.

**Table 2:** Immunohistochemical characteristics of the neoplastic cells in our HPN, perineurioma, and cellular neurothekeoma

	**Our case (HPN)**	**Perineurioma**	**Cellular neurothekeoma**
MiTF	+	**-**	+
NKI/C3	+	**-**	+
PGP.5	+	+	+
NSE	+	**-**	+
Glut-1	+	+	**-**
CD 10	+	+	**-**
CD99	+	+	**-**
S-100protein	**-**	**-**	**-**

## Competing interests

The authors declare that they have no competing interests.

## Authors’ contributions

SY and MH participated in conception of the idea and writing of the manuscript. SY, SK, AN, HN, YS, and MH performed the pathological and immunohistochemical interpretation of the tumor tissue. All authors have read and approved the final manuscript.
